# Understanding health care price variation: evidence from Transparency-in-Coverage data

**DOI:** 10.1093/haschl/qxaf011

**Published:** 2025-01-21

**Authors:** Christopher Whaley, Nandita Radhakrishnan, Michael Richards, Kosali Simon, Benjamin Chartock

**Affiliations:** School of Public Health, Brown University, Providence, RI 02903, United States; School of Public Health, Brown University, Providence, RI 02903, United States; Jeb E. Brooks School of Public Policy, Cornell University, Ithaca, NY 14853, United States; O’Neill School of Public Affairs, Indiana University, Bloomington IN 47405, United States; Department of Economics, Bentley University, Waltham, MA 02452, United States

**Keywords:** costs and spending, price transparency, commercial insurance, health care markets

## Abstract

Competition in health care markets should lead to lower prices and less dispersion, with consumer choice as the driving mechanism. Several studies document price variation, suggesting room for improvement; however, they relied on selected data from insurers who provide access to data, limiting generalizability. We document the nature of price variation in the private US market across geography, payer, and provider by leveraging a new dataset, implementing a descriptive analysis using the most comprehensive data available: Transparency-in-Coverage. We measured health care prices in 3 ways: percentile distribution prices for common services, state-level and insurer-level facility fee price indices, and regression-adjusted mean inpatient and outpatient prices. Variation is large: the mean facility fee for a foot X-ray, for example, is $86 at Anthem and $190 at UnitedHealth. Pricing does not appear to be uniform; there is just 22% correlation between an insurer's inpatient price and outpatient facility price. And there is little difference in ordering of high-price states depending on alternative measures, such as relative to Medicare. Results suggest greater consideration of policies to address high and variable prices for US health care.

## Introduction

In an economic market, competition between firms should lead to lower prices and higher quality services when information is transparent. However, US health care markets are substantially higher priced than in comparable countries, largely due to high and variable prices for the approximately 180 million Americans with commercial insurance.^1,2^ Because prices are negotiated in nontransparent ways, patients, providers, and policy makers have little ability to compare prices, which limits patient ability to price-shop, employer and purchaser ability to design efficient health care benefits, and policymaker and regulator ability to effectively ensure market competition.

Although tools have emerged to give patients information on prices,^3^ these tools have low use rates and have not meaningfully changed patient behavior or pricing dynamics.^4,5^ Likewise, while several studies have documented price variation, these studies relied on selected data from insurers that provide access to medical claims data, potentially limiting the generalizability of the findings.^6,7^ Data used in existing studies do not commonly allow for the identification of specific insurers or providers. The lack of transparent information on health care prices limits the ability of purchasers to both monitor prices negotiated on their behalf and inform benefit design decisions, as well as the ability of policymakers and regulators to monitor health care markets.

However, recent federal policies greatly expand access to price transparency data. The 2020 Transparency-in-Coverage (TiC) Executive Order requires insurance companies to post negotiated rates for all commercially insured contracts.^8^ Although released in July 2022, these data have not been widely used, largely due to data structure and complexity. One major dimension of this complexity is that the rule requires prices for a substantial number of services that are rarely, if ever, provided. This leads to inflated data size and potentially introduces bias into TiC price measures––referred to as “zombie rates” in nontechnical settings.^9-11^ Efforts to improve TiC data usability and access have been a recent policy focus.^12,13^ Existing studies have used the TiC data from a single insurer, Humana, and compared the TiC data with claims data, finding them to be comparable.^14,15^ Other studies have examined the processes to use the TiC data and identified potential improvements.^16,17^

To improve usability of the TiC data and address data complexity, the TiC data used in this study links insurer-posted data with claims-based information from approximately 270 million people. Relative to claims-based data used in previous studies, the TiC data allow for plan and provider-identifiable price information. We use these data to illustrate the range in prices by both insurer and geographic market. The results of this study both illustrate how these novel data can be used to inform health care pricing analyses and policies, as well as provide a detailed examination of health care prices. This paper contributes to studies on health care prices and on the use of novel data by constructing a national price index for each major insurer across a variety of procedures. Until this point, restrictions on identifying prices by provider or insurer limited the ability of such analyses to inform policy and purchasing decisions. The results of this paper shed light on the wide variation in health care prices and show how these novel data can be used to examine US commercial health care prices in a more comprehensive manner than previously possible.

## Data and methods

### Transparency in coverage regulation

The TiC regulation follows a 2019 Trump administration executive order on improving price transparency. The Centers for Medicare and Medicaid Services (CMS) has described the regulation as having the “goal of bringing greater competition to the private health care industry.”^11^ The largest component of the TiC regulation requires group health plans and health insurance issuers in the individual market to post online machine-readable files including negotiated rates for all covered services and in-network providers. A notable characteristic of the regulation is steep fines for noncompliance. Unlike the earlier hospital transparency pricing rule, where fines for noncompliance were negligible, the insurer-based TiC rule imposes heavy fines on insurers who do not post their prices, $100 per enrollee per day that an insurer is noncompliant. As a result, there has been nearly full participation by insurance companies with respect to machine-readable files, which is not the case for hospital-posted transparency files.^18^

However, aggregating these universal pricing data across payers and providers has proven difficult for researchers, due to both obfuscation by payers and administrative challenges in roll-out and standardization of the data. Recently, a small number of private entities specializing in TiC have arisen to aggregate and clean these data, providing them to the business community as well as academic researchers. This feature is by design. Policymakers' objectives were to spur innovative use of these data by the private sector related to price-shopping tools. We rely on this type of data in this article.

### Data sources

To analyze the TiC data, we use data from a third-party source, Clarify Health, under a data use agreement. Clarify Health aggregates TiC data from monthly updates of insurer TiC data postings. An existing limitation of many TiC data postings are the inclusion of “zombie rates” for providers that do not perform services but are listed in an insurer's pricing files.^10^ These rates both lead to inaccurate pricing, by including price observations for providers unlikely to perform a procedure, and inflate the size of the data, making use more difficult. Many insurers also post price information for overlapping plans (eg, individual self-funded, employer-sponsored plans that use the same insurer as a third-party administrator have separate pricing files) and networks. Due in large part to these inclusions, the full set of posted TiC data across all insurers includes over 1 trillion price observations in any given month. The combined TiC monthly file sizes are over 1 petabyte.

To address this data challenge, Clarify links TiC data to a 100% sample of Medicare fee-for-service (FFS) enrollees and commercial insurance claims data. Collectively, these claims data cover approximately 270 million people.^19^ We limited TiC prices to providers with billed claims for each relevant procedure. Unfortunately, the claims data do not identify insurers, and so we were unable to link to provider/procedure prices for specific insurers. In cases where a provider performs a service for patients covered by 1 insurer but not for other insurers, the TiC data are unable to distinguish between prices that represent insurer-specific provider volume.

We limited our sample to prices from the 5 national insurers: Blue Cross Blue Shield (BCBS), UnitedHealth Group, Cigna, Aetna, and Humana. These insurers account for the majority of covered enrollees and are also likely different in many unobservable ways from smaller fringe insurers. Due to their joint participation in the BCBS Association, the Clarify data group the state-level BCBS plans and Elevance (formerly Anthem) under 1 plan grouping. The “BUCAH” plans account for 78% of commercial insurance volume.^20^

### Health care price measures

With these data, we measured health care prices using 3 approaches. First, separately for each national insurer and across all combined insurers, we calculated mean, 25th percentile, median, and 75th percentile prices for common services, which were identified according to Current Procedural Terminology (CPT) and Diagnosis Related Group (DRG) codes. These procedures included professional services (established patient office visit [CPT code 99213] and emergency department visit [CPT code 99285], outpatient procedure facility fees), foot X-ray (CPT code 7360) and diagnostic colonoscopy (CPT code 45378), and inpatient facility fee (major joint replacement without complications [DRG 470] and heart failure and shock with complications [DRG 291]). These measures provide a high-level and interpretable view into how prices vary both across and within national insurers for common services.

Second, we expanded our price measures beyond these common services and constructed state-level and insurer-level facility fee price indices for inpatient and outpatient services.^21-24^ These indices measure the weighted-average price within a state, after weighting for the relative contribution of each procedure's price and volume to overall spending. The full description of this index construction is further described in the Supplementary Appendix. These price indices measure percentage differences from national weighted-average prices.

Finally, while the price index allows for an aggregation of prices, it does not address the contribution of procedure composition, insurer, or geographic market factors to commercial price variation. We estimated multivariable linear regressions with fixed-effects controls for procedure, state, and insurer. Thus, these regressions measure price differences, after adjusting for time-invariant procedure, geographic market, and insurer characteristics. We used the predicted values from these regressions to calculate the regression-adjusted mean inpatient and outpatient price for each state and insurer. These regressions were separately estimated for inpatient and outpatient procedures.

While the TiC price data contain prices at the procedure-provider-insurer level, 1 limitation is that the posted data do not include measures of volume, thus limiting the ability to compare differences in spending across providers. As addressed above, Clarify data merge provider-procedure volume data. In sensitivity tests, we used each provider's procedure-specific commercial insurance volume to construct the state-level share of procedures performed by each provider (we computed state-specific price indices). We used these provider volume shares as a weight in our regressions. In additional sensitivity tests, we likewise accounted for differences in insurer data contribution across markets by similarly weighting insurer market share within each state.

For these regressions, we used both absolute dollar-unit prices and commercial prices relative to Medicare payment rates for the same provider and procedure as our dependent variable. Inpatient commercial prices relative to Medicare allow for the adjustment of operating cost differences across regions and procedure input intensity, as well as adjusts for provider costs that may impact operating costs, such as graduate medical education and care provision to Medicaid and uninsured patients.^7^

## Results

### Price variation among common services, within and across insurers

Table 1 presents mean and 10th, 25th, 50th, 75th, and 90th percentile prices for common services for each of the national insurers, as well as national averages. Prices vary considerably, both within and across procedures. For common evaluation and management office visits (CPT 99213), mean insurer prices range from $82 for Aetna to $115 for UnitedHealth. Across all payers, the mean reimbursement rate is $104. Median prices are approximately 10% lower than mean prices, indicating upward-dispersion in reimbursement rates. Within insurers, the ratios of the 75th and 25th percentile prices range from 1.2 for Aetna, indicating that 75th percentile prices are 20% higher than 25th percentile prices, to 1.5 for UnitedHealth. Similar levels of variation exist for emergency department professional fees. Insurer-level mean prices range from $242 for Aetna to $321 for Cigna. Across all insurers, the 75th percentile payment rate is 1.7 times the 25th percentile.

**Table 1.** Common-procedure price variation across national insurers.

	Mean	25th percentile	Median	75th percentile	Ratio, 90th/10th percentile
Established patient office visit (CPT 99213) professional fee					
Aetna	$82	$74	$77	$86	1.29
Blue Cross Blue Shield	$102	$82	$93	$113	1.84
Cigna	$108	$84	$98	$119	2.00
Humana	$111	$89	$101	$121	1.89
United Healthcare	$115	$87	$106	$131	2.14
All payers	$104	$81	$93	$117	1.98
Emergency department (CPT 99285) professional fee					
Aetna	$242	$158	$169	$198	3.30
Blue Cross Blue Shield	$287	$225	$266	$331	2.13
Cigna	$321	$207	$259	$374	3.34
Humana	$263	$195	$229	$287	2.32
United Healthcare	$290	$213	$263	$337	2.40
All payers	$282	$194	$247	$322	2.63
Foot X-ray (CPT 73630) facility fee					
Aetna	$123	$85	$109	$149	2.78
Blue Cross Blue Shield	$162	$98	$132	$208	3.35
Cigna	$163	$87	$127	$207	4.01
Humana	$86	$64	$74	$90	2.00
United Healthcare	$190	$137	$182	$195	2.77
All payers	$152	$91	$128	$194	3.40
Diagnostic colonoscopy (CPT 45378) facility fee					
Aetna	$2686	$1147	$2316	$3671	6.58
Blue Cross Blue Shield	$1847	$965	$1321	$2252	4.63
Cigna	$1481	$850	$1386	$2031	3.43
Humana	$2166	$900	$1507	$2993	6.78
United Healthcare	$2393	$1065	$1967	$3283	5.82
All payers	$2352	$1036	$1826	$3224	6.00
Major joint replacement without complications (DRG 470) facility fee					
Aetna	$32 499	$21 346	$29 735	$39 873	3.08
Blue Cross Blue Shield	$29 779	$20 936	$27 640	$36 775	2.92
Cigna	$31 072	$20 866	$27 463	$37 882	2.97
Humana	$27 295	$19 475	$26 280	$33 100	2.73
United Healthcare	$31 748	$22 766	$29 813	$38 595	2.71
All payers	$31 093	$21 472	$28 789	$38 010	2.88
Heart failure and shock with complications (DRG 291) facility fee					
Aetna	$21 059	$14 614	$19 261	$26 556	2.92
Blue Cross Blue Shield	$18 125	$12 671	$16 237	$22 098	2.74
Cigna	$20 247	$13 703	$18 359	$24 351	2.85
Humana	$18 003	$12 748	$17 244	$21 161	2.76
United Healthcare	$19 983	$14 120	$18 572	$24 425	3.00
All payers	$19 588	$13 554	$17 983	$24 047	2.90

The table presents mean and 10th, 25th, 50th, 75th, and 90th percentile prices for common services for each of the national insurers, as well as national averages.

Abbreviations: CPT, Current Procedural Terminology; DRG, Diagnosis Related Group.

For outpatient procedures, X-ray facility fee mean payments range from $86 for Humana to $190 for UnitedHealth. Across all insurers, the ratio of the 75th and 25th price percentiles is 2.1. Prices are higher and more variable for diagnostic colonoscopy facility fee payments. Mean prices range from $1481 for Cigna to $2686 for Aetna. Across all insurers, the 75th–25th percentile price ratio is 3.1, but ranges from 2.3 for BCBS plans to 3.3 for Humana.

Among our 2 selected inpatient procedures, mean joint replacement facility fee prices range from $27 295 for Humana to $32 499 for Aetna. Across all national insurers, the 75th price percentile ($38 010) is 1.8 times the 25th price percentile ($21 472). Similar ranges exist for heart failure facility fees, with the 75th percentile ($24 074) being 1.8 times larger than the 25th percentile ($13 554). Mean insurer-specific prices range from approximately $18 000 for BCBS and Humana to $21 059 for Humana.

### Price variation among national insurers

**Figure 1.**
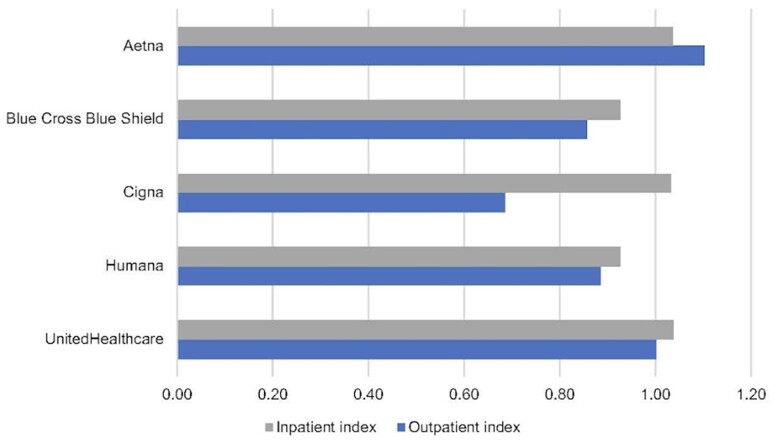
Price index by insurer showing differences in indexed inpatient and outpatient prices among national insurers.

Figure 1 presents differences in indexed inpatient and outpatient prices among national insurers. Relative to the national average (1.0), insurer prices for inpatient services range from 7% below average for BCBS plans and Humana, to 4% higher for Aetna and UnitedHealth. Insurer prices have larger variation for outpatient services, with prices ranging from 31% below the national average for Cigna to 10% higher for Aetna. There is little correlation between an insurer's inpatient and outpatient price index, with a correlation coefficient between the 2 indices of 0.23.

### Regression-adjusted price variation across states

**Figure 2.**
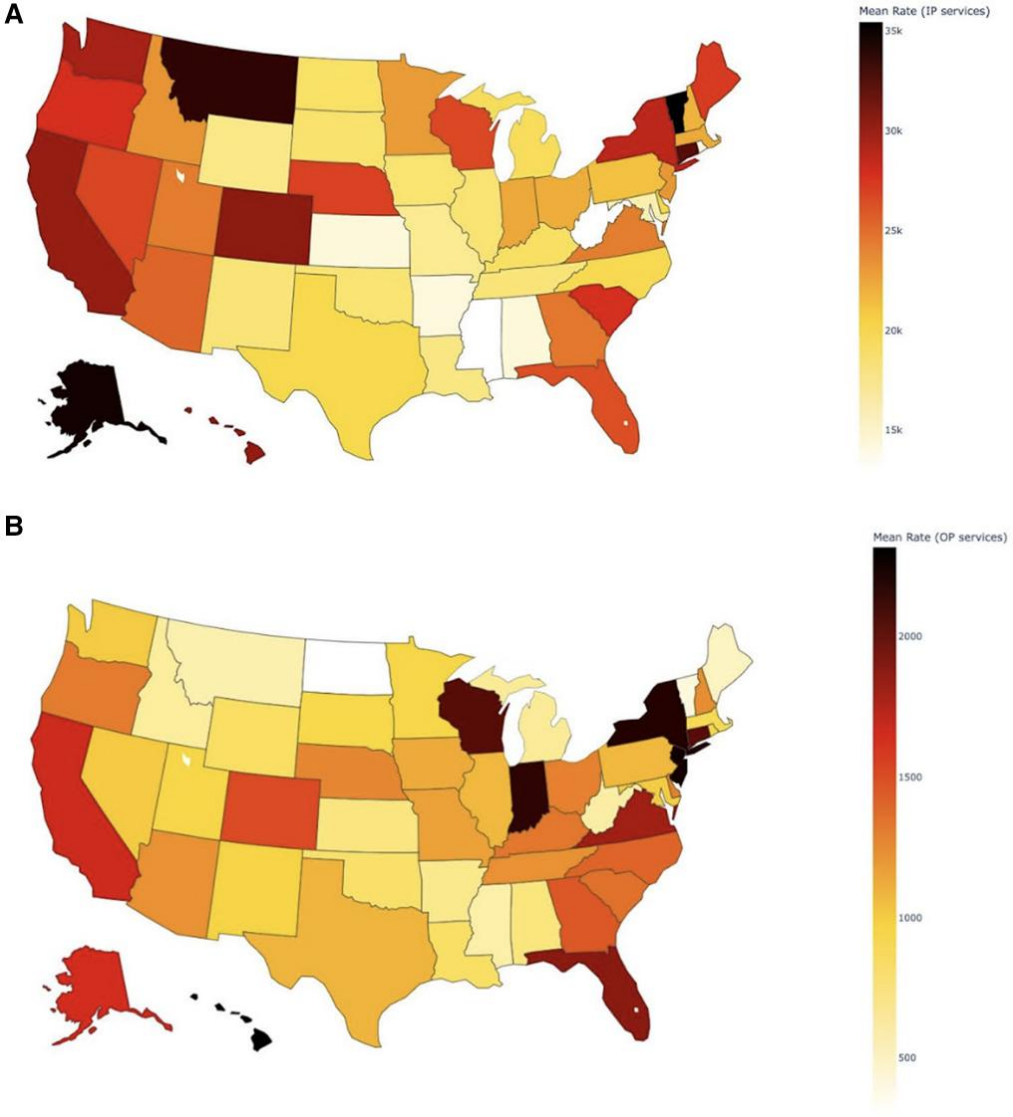
State-level variation in commercial insurance prices showing regression-adjusted state-level prices for inpatient (IP) (A) and outpatient (OP) (B) services.

Figure 2 maps regression-adjusted state-level prices for inpatient (panel A) and outpatient (panel B) services. After adjusting for procedure composition and insurer differences within states, mean inpatient prices range from $35 435 in Vermont to $12 917 in Mississippi. Regression-adjusted prices for outpatient services range from $2317 in Hawaii to $294 in North Dakota. Thus, across states, regression-adjusted commercial insurance prices vary by 174% for inpatient services and 688% for outpatient services, respectively. Using this approach, the state-level correlation between prices for inpatient and outpatient services is 0.42.

**Figure 3.**
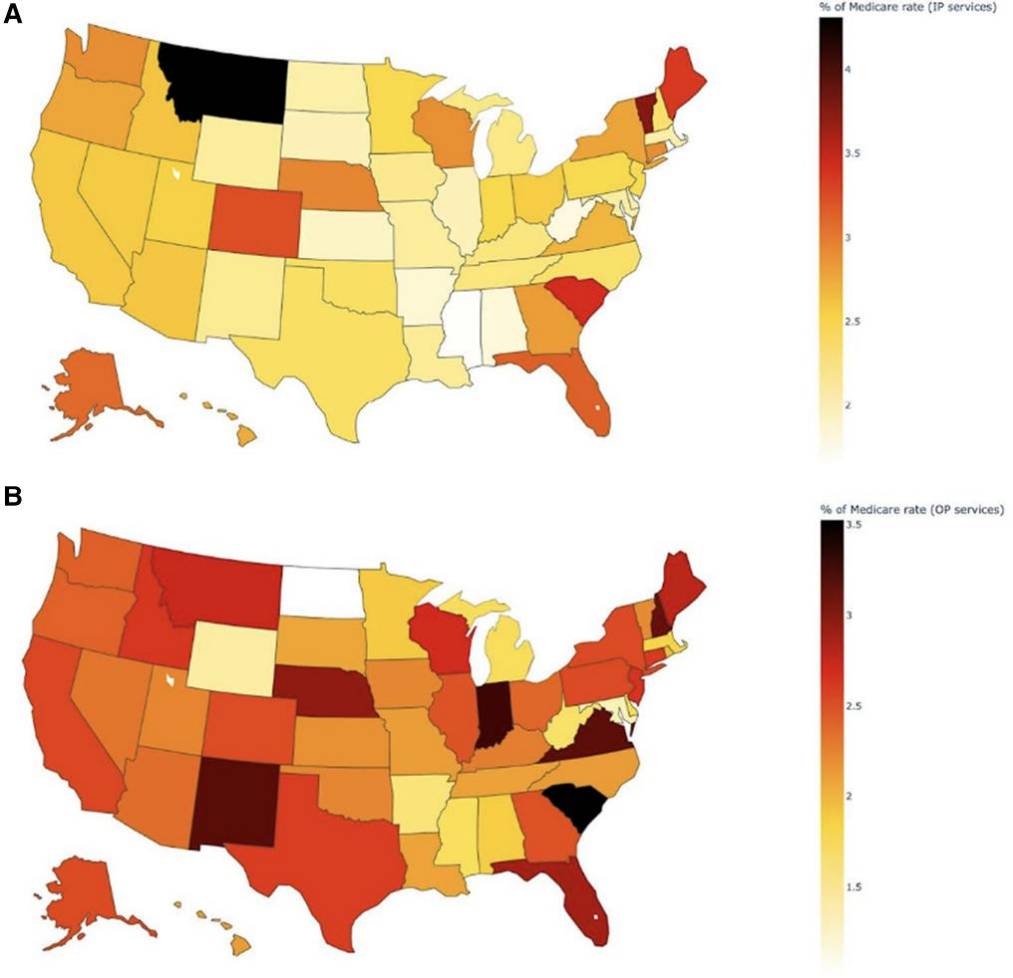
State-level variation in commercial insurance prices showing regression-adjusted state-level prices for inpatient (IP) (A) and outpatient (OP) (B) services, measured as a percentage of Medicare.

Figure 3 presents similar differences, but measured as a percentage of Medicare. As a percentage of Medicare, inpatient prices range from 431% in Montana to 163% in Rhode Island and outpatient prices range from 352% in South Carolina to 100% (eg, the same as Medicare rates) in North Dakota. As a percentage of Medicare, state-level regression-adjusted prices vary by 164% for inpatient services and 252% for outpatient services. As a percentage of Medicare rates, the state-level correlation between inpatient and outpatient service prices is 0.56.

**Figure 4.**
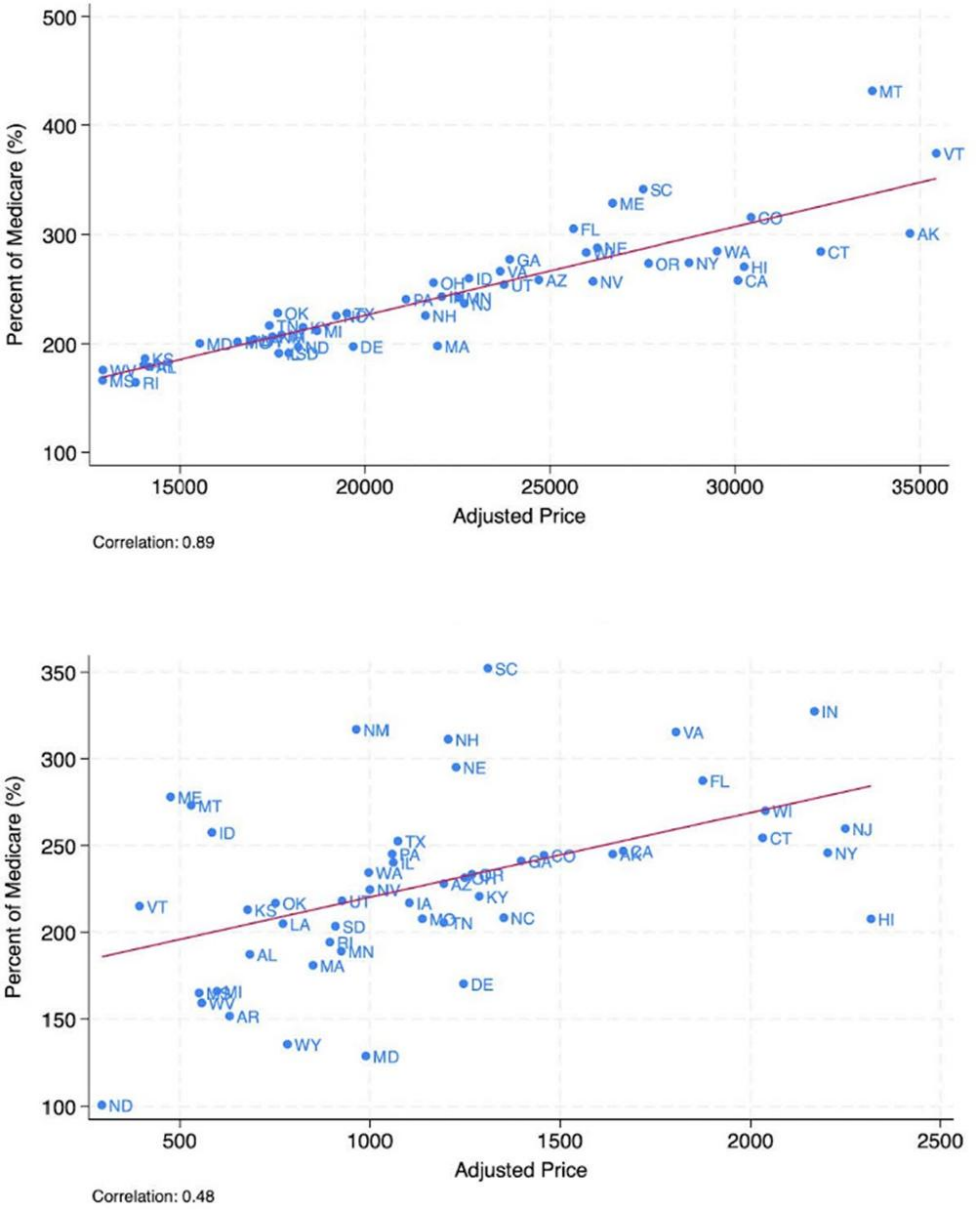
Relationship between state-level prices when measured as a percentage of Medicare and adjusted prices.

Figure 4 plots the regression-adjusted relationship between state-level prices when measured as a percentage of Medicare and adjusted prices. For inpatient procedures, the correlation between state-level regression-adjusted prices, when measured as a percentage of Medicare or in dollar terms, is 88%. For outpatient procedures, choice of price measures yields similar results as well. The correlation between outpatient facility prices, when measured in either dollar terms or as a percentage of Medicare, is 48%.

### Sensitivity analyses

Appendix Figures S1 and 2 show state-level differences in regression-adjusted prices weighted by provider commercial service volume weights and insurer-specific regression weights, respectively. For inpatient services, this approach yields similar results as the unweighted prices. However, accounting for differences in provider volume is more of a consideration for inpatient services. The correlations between regressions that do not weight, weight by insurer volume, and weight by provider volume are approximately 78% for outpatient services and 39% for inpatient services.

Appendix Figure S3 uses the index approach to measure state-level inpatient and outpatient prices. Relative to the national average (1.0), inpatient facility prices range from 0.53 in Maryland to 1.5 in Vermont. Likewise, outpatient prices range from 0.42 in North Dakota to 1.42 in Indiana. The correlation between a state's price index for inpatient and outpatient facility prices is 0.48.

## Discussion

Per-capita health care spending in the United States is approximately twice that of comparable countries, largely due to high and variable prices, particularly for those with commercial insurance.^25^ Despite their importance, commercial insurance prices are opaque and nontransparent, even though several policy and technology efforts have attempted to improve price transparency. This paper leverages newly released data on all commercial insurance prices in the United States to document a more comprehensive evaluation of price variation than currently exists. Importantly for this study, the TiC data allow for comparisons of prices between individual and named providers and insurers, which is uncommon among existing studies.

Consistent with other studies, we found large variation in health care prices. However, unique to this study was the ability to include price measures from all national insurers and geographies, and to list prices by specific insurer. Using this new and comprehensive source of national pricing data, we had 3 main findings. First, prices vary widely, both within and across insurers. Among national insurers, prices for common procedures vary by 50% to over 200%. Insurer pricing does not appear to be uniform, with just a 22% correlation between an insurer's inpatient and outpatient facility prices for common services. Second, mean prices across geographic markets vary widely across states, even after adjusting for insurer and procedure differences. Across states, the 75th percentile regression-adjusted price is 1.48 times larger than the 25th percentile price for inpatient services, and 65% larger for outpatient services. Finally, there is little difference in the relative ordering of state-level prices when using alternative measures of commercial prices or analytic approaches, suggesting that commercial price variation measures are robust to alternative analyses.

This paper is not without limitations. Most notably, while we document commercial price variation, we do not identify sources of price variation. If higher prices are tied to, or even lead to, higher-care quality, then policies that focus on reducing prices may harm patient outcomes. However, existing evidence shows a minimal link between price and quality.^26-29^ If prices are due to provider consolidation, either through hospital mergers, hospital acquisition of physician practices, or, as some have recently hypothesized, private equity acquisition, then the results of this study can inform the importance of stronger regulatory oversight of health care consolidation.^30^ At the same time, this study identifies high and variable prices among national insurers, which should have substantial negotiation leverage. Future studies should examine the contracting and network design practices that contribute to these prices and their variation. Relatedly, we did not account for potential differences in procedure coding or grouping (eg, bundled payments) across insurers and providers. While we weighted by a provider's entire commercial volume, we lacked insurer-specific provider and procedure claim volume. Finally, while the TiC data are a new and national source of price transparency data, due to file size and construction they are not currently widely available without accessing through a private vendor.

Despite these limitations, the TiC data used in this study are likely the most comprehensive source of data on prices negotiated between commercial insurers and providers. It is challenging to find a similar example of such large price variation for other goods and services, particularly those that account for such a large magnitude of spending. In addition, US health care markets are a focus of regulatory oversight and many policy initiatives. Despite these efforts, commercial prices vary widely, with large and potentially unexplained variation in prices between states. As these data become more widely used by insurers, purchasers, and policymakers, research should examine the impacts of policies that use these data—such as those that direct patients to lower-priced providers. Removing potentially unwarranted price variation creates a large opportunity for reducing health care spending, often with improvements in patient health outcomes.

## Supplementary Material

qxaf011_Supplementary_Data
