# Supplementary material for: Understanding health care price variation: evidence from Transparency-in-Coverage data
Source: Health Aff Sch. 2025 Jan 21;3(2):qxaf011. doi: 10.1093/haschl/qxaf011 (PMC11798183; doi:10.1093/haschl/qxaf011)

Supplemental Appendix

Table of Contents

1. Methods
2. Sensitivity of Results to Alternative Weighting Approaches
3. Analysis of State Price Rankings
4. Comparisons to Claims Based Approaches

1. Methods

Key Price Index Components

Included Services

For a DRG code to be a candidate code for inclusion in the price index, it must be present with sufficient volume. To determine this, we used the State of North Carolina's Department of Health and Human Services Top 100 Reported Diagnostic Related Groups (DRGs) by volume.[1] The DRG must also occur for all payers. 92 DRG codes met this criteria. The set of these codes was chosen to include a range of typical inpatient services. For our outpatient price index, we created a list of 23 CPT codes encompassing a range of hospital outpatient services from scans (CT and X-Ray) to tissue exam by pathologist to diagnostic colonoscopy. The full list of included outpatient and inpatient codes is below. Similar to the inpatient code list, the outpatient codes list comes an existing study that develops a price index for outpatient services.[1]

Table S1: Billing Codes Used to Construct Price Measures

**Outpatient CPT Codes:**	**Inpatient DRG Codes**:			
43239	57	247	390	690
45378	64	253	391	698
45380	65	280	392	743
45385	66	281	394	789
71046	69	282	418	790
71250	100	286	419	791
71260	101	287	439	792
72100	175	291	440	793
73030	176	292	460	794
73502	177	293	473	795
73630	178	308	481	812
74177	189	309	483	847
76536	190	310	552	853
76642	191	312	603	871
76700	192	313	621	872
76830	193	314	637	881
76856	194	329	638	882
77080	195	330	639	885
86850	202	331	640	897
88305	203	372	641	917
93005	207	377	682	918
93017	208	378	683	948
93306	246	389	689	949

Service Prices

For the TiC rates used in the calculation of the price indices, we employed the following inclusion strategy: we limited our sample to institutional rates from the above inpatient and outpatient services, and to providers with billed commercial claims for each procedure.

[1] https://info.ncdhhs.gov/dhsr/ahc/hb834/drg100.html

Additionally, we restricted our sample to rates that are between 75% and 1000% of the Medicare rate, to eliminate outliers.

Service Quantities
When including quantities in price indices, we used the number of commercial services billed for the procedure-provider pair. In addition to aggregating TiC data, Clarify collects medical claims data from commercial insurers and Medicare. These claims data include information on approximately 270 million individuals. Unfortunately, the procedure volume data do not allow for identification of individual insurers.

Hospital Index Computation
Following existing studies, we constructed price indices in the following manner.[1–4] For each insurer i, procedure k (where k can be a DRG or CPT) and state g, we take the following two steps. First, we define procedure weights, the share that each procedure contributes to spending:

$$w_k = \frac{price_k \cdot q_k}{\sum^K price_k \cdot q_k}$$

where $price_k$ is the mean procedure price, q_k is the total commercial procedure volume. Then, we define separately three indices for insurer and state price index.

The insurer index is

$$index_i = \sum^K \frac{price_{ik}}{price_k} w_k$$

i.e., an index of 1.04 indicates an insurer is 4% more expensive than the national average.

The state index is

$$index_g = \sum^K \frac{price_{gk}}{price_k} w_k$$

Where g represents each state, and an index of 1.04 indicates a state is 4% more expensive than the national average.

Multivariate Regressions

In addition to the price index, we estimate a multivariate linear regression of the form

$$price_{kt} = \alpha + \beta_1 procedure_k + payer_p + state_t$$

Where $procedure_k$, $payer_p$ and $state_t$ represent procedure (from Table S1), insurer and state fixed effects, respectively. We obtained the predicted values of this regression to examine price variation, after adjusting for procedure and market (state)-level differences.

2. Sensitivity of Results to Alternative Weighting Approaches

This study uses newly-released "Transparency-in-Coverage" (TiC) data on insurer prices to examine price variation among commercial insurers in the United States. For all insurers, these data contain price lists of procedure- and provider-specific prices. While a comprehensive set of prices, these data differ from other data sources that have been used to examine these questions.

Most notably, unlike medical claims data, they do not contain measures of provider-specific volume, limiting the ability to account for differences in provider quantities. In other words, the TiC data treat each provider as an equal unit, when some providers may perform more or less than the market-level average number of services. Likewise, these data do not account for differences in the representativeness of an insurer's population within a state.

To account for these limitations, we test the sensitivity of our regressions by estimating similar regressions that 1) use the volume of a provider's commercial service volume for a given procedure in the Clarify data as regression weights, and 2) use the insurer's share of commercial insured population in a state as a weight.

We find a 39% correlation for inpatient prices and 77% correlation for outpatient prices between the unweighted regressions and regressions that weight by each provider's state-level market share (Figure 1). Similarly, we find a 61% correlation for inpatient prices and 78 % correlation for outpatient prices between the unweighted regressions and regressions that weight by the insurer's share of commercial insured population in a state (Figure 2).

3. Analysis of State Price Rankings

In addition to reporting index-based and regression-adjusted prices across states, we used the index computation procedure outlined above to rank states based on price, in order from least to most expensive. As reported in Figure 3, the least expensive states for inpatient services are Maryland, Mississippi and Alabama, while the most expensive states are Connecticut, Alaska, and Vermont. The corresponding outpatient rankings are North Dakota, Maryland and Wyoming (least expensive), and South Carolina, Connecticut, and Indiana (most expensive).

4. Comparisons to Claims-Based Approaches

As a final comparison, we compared the state-level percent of Medicare estimates from the TiC data with claims-based percent of Medicare, which were derived from a national study of U.S. hospitals.[5] For outpatient services, the TiC data contain both hospital-based, as well as non-hospital (e.g., imaging center, laboratory test site, ambulatory surgical center) sites of care, while the claims data are restricted to hospital outpatient department observations. As shown in Figure 4, we find a 31% correlation for inpatient services, and a 39% correlation for outpatient services.

Figure 1: Relationship between unweighted regressions and regressions that weight by provider's state-level market share

Panel A: Inpatient Facility

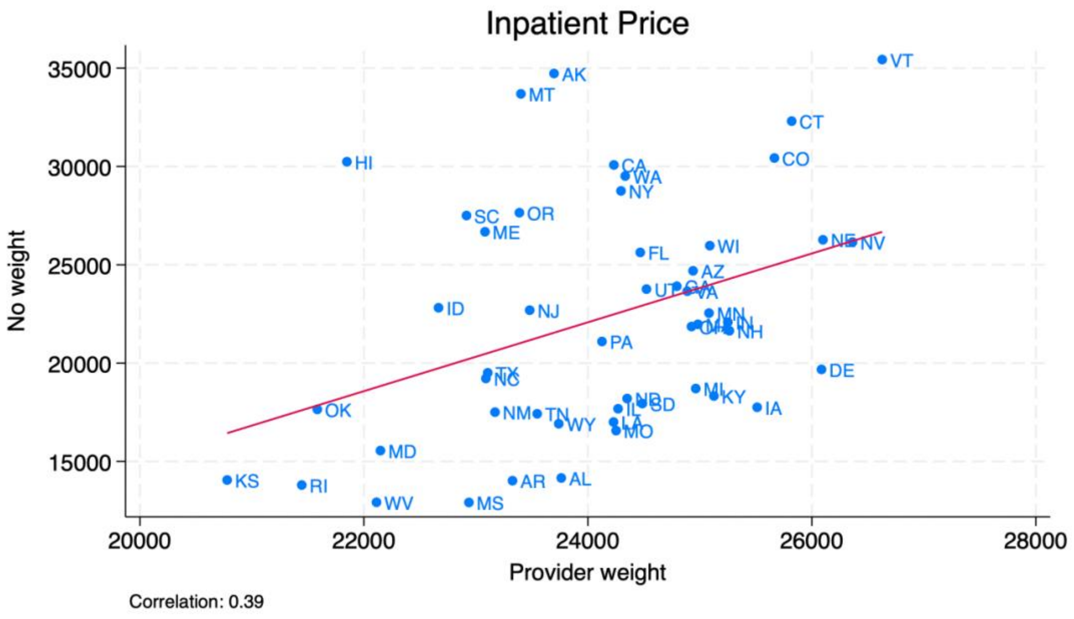

Panel B: Outpatient Facility

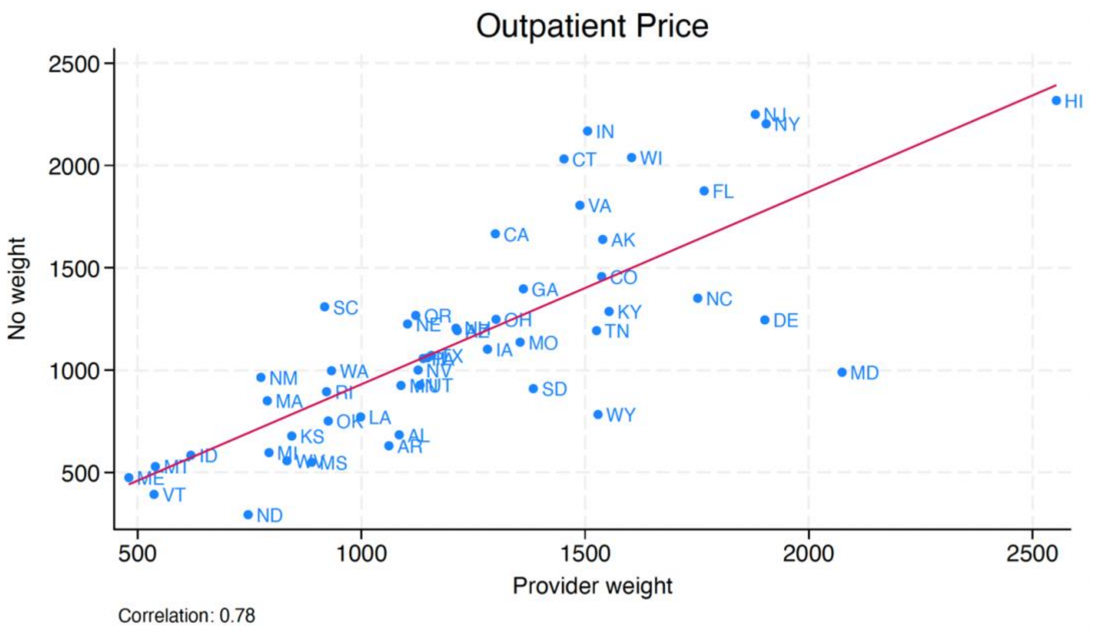

Figure 2: Relationship between unweighted regressions and regressions that weight by insurer's state-level market share

Panel A: Inpatient Facility

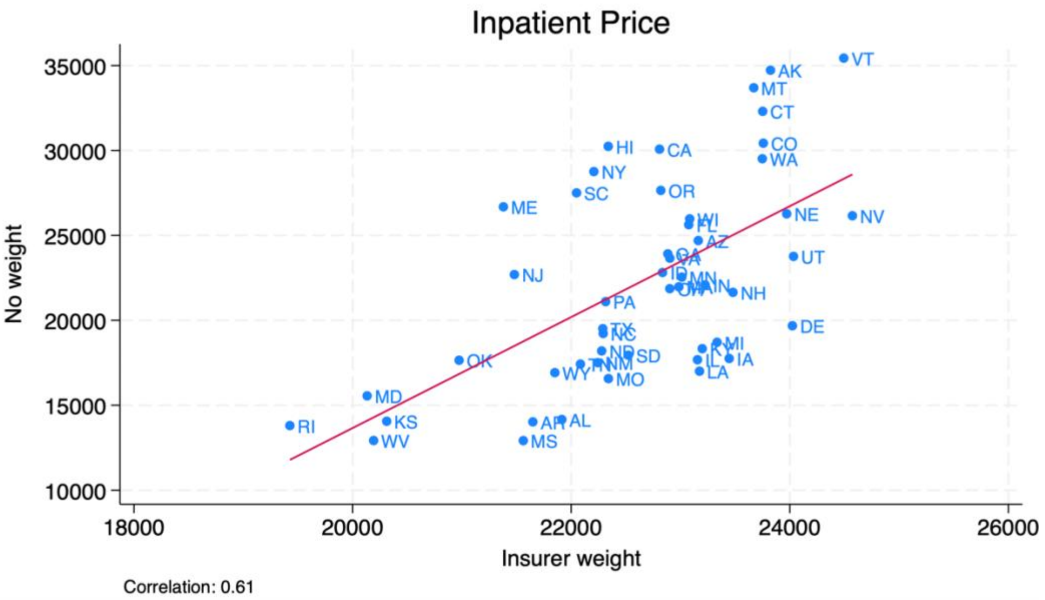

Panel B: Outpatient Facility

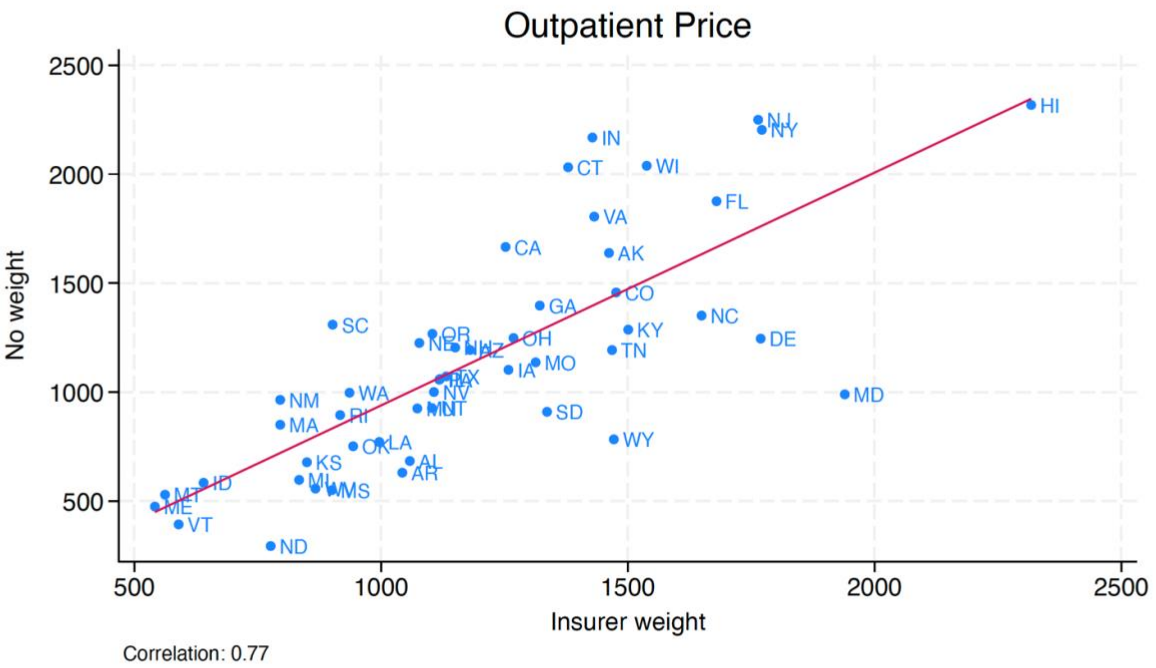

Figure 3: State-Level Variation in Market Price Index

Panel A: Inpatient Facility

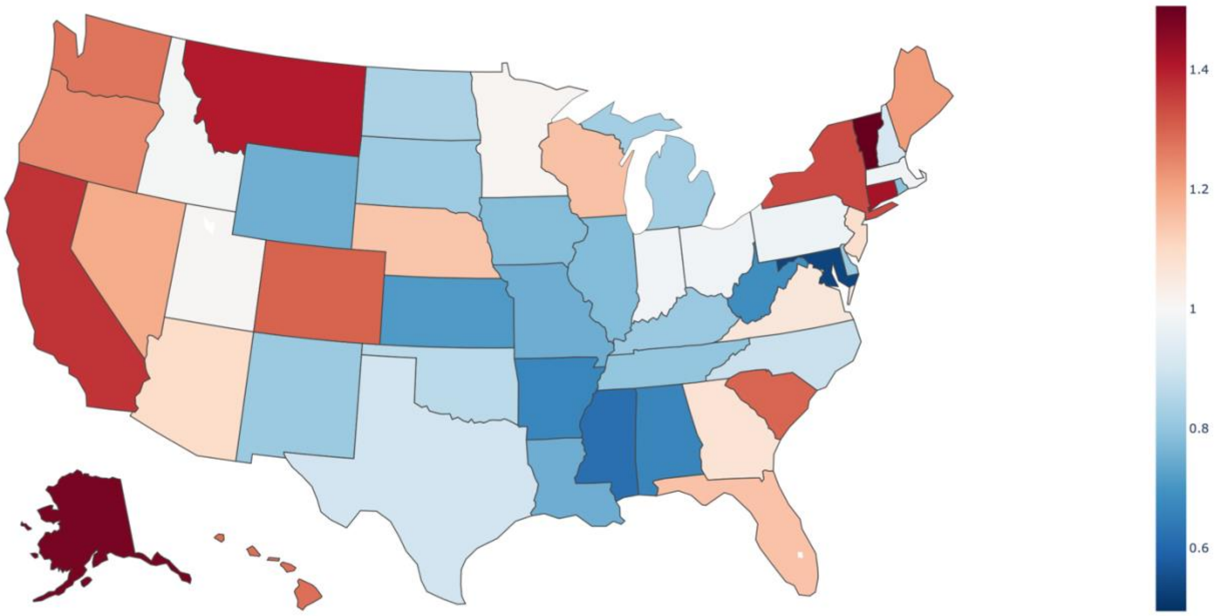

Panel B: Outpatient Facility

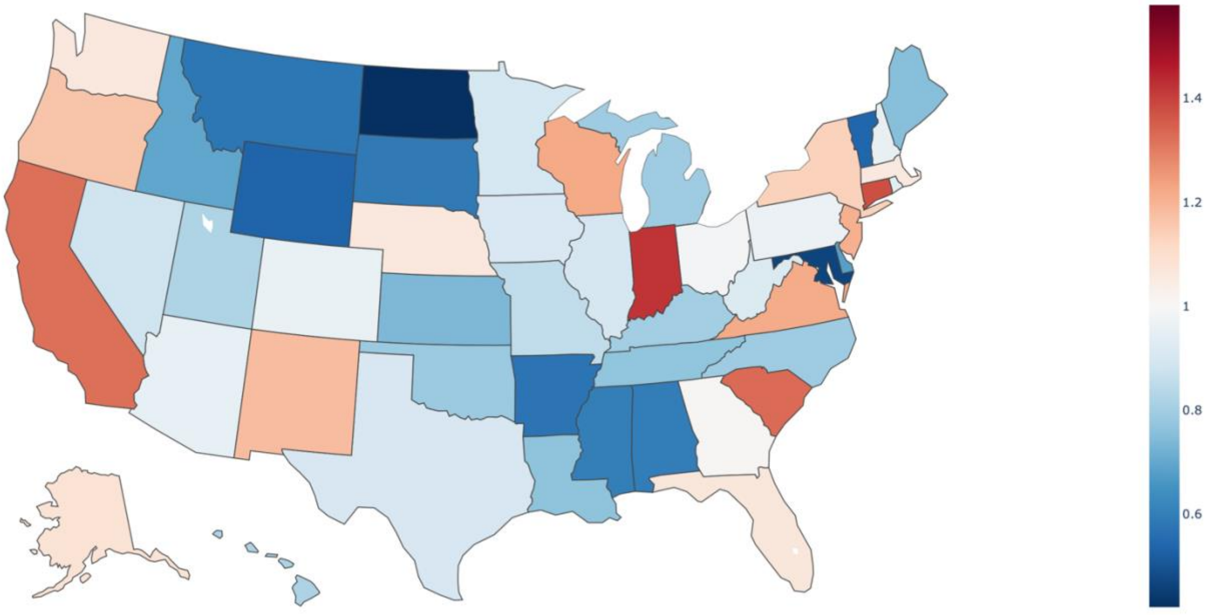

Supplement: qxaf011_Supplementary_Data [file qxaf011_supplementary_data.zip › Supplemental Appendix.pdf]
